# Secondary dementia due to Lyme neuroborreliosis

**DOI:** 10.1007/s00508-018-1361-9

**Published:** 2018-07-25

**Authors:** Wolfgang Kristoferitsch, Fahmy Aboulenein-Djamshidian, Julia Jecel, Helmut Rauschka, Michael Rainer, Gerold Stanek, Peter Fischer

**Affiliations:** 1 Karl Landsteiner Institute for Neuroimmunological and Neurodegenerative Disorders, SMZ-Ost-Donauspital, Langobardenstr. 122, 1190 Vienna, Austria; 2 Neurological Department, SMZ-Ost-Donauspital, Vienna, Austria; 3 Neurological Department 2, NKH Rosenhügel, Vienna, Austria; 4 Psychiatric Department, SMZ-Ost-Donauspital, Vienna, Austria; 5 Karl Landsteiner Institute for Memory- and Alzheimer Research, SMZ-Ost-Donauspital, Vienna, Austria; 6 Institute for Hygiene and Applied Immunology, Medical University of Vienna, Vienna, Austria

**Keywords:** Lyme borreliosis, Lyme disease, Cognitive impairment, Antibiotic treatment, Normal pressure hydrocephalus

## Abstract

Dementia-like syndromes are rare manifestations of Lyme neuroborreliosis. The clinical patterns are summarized using our own cases and case reports from the literature, which were diagnosed as definite Lyme neuroborreliosis according to the European guidelines. The cases disclose signs of subcortical dementia that occur more rapidly than in patients suffering from primary dementia. Gait disturbances early in the disease course is another frequently observed characteristic feature. The response to 2–4 weeks of antibiotic treatment with ceftriaxone was excellent. There were no indications for a prolonged antibiotic treatment. It is essential to be aware of this manifestation of Lyme neuroborreliosis, because early antibiotic treatment will prevent permanent sequelae that may occur throughout the further course of the untreated disease.

## Introduction

Dementia as a dominant symptom of meningoencephalitis in Lyme neuroborreliosis (LNB) is extremely rare. Only few reports on definite cases with LNB exist, when guidelines of the European Federation of Neurological Societies (EFNS) on diagnosis of LNB are applied [1]; however, it has been argued that dementia-like syndromes associated with Lyme borreliosis (LB) or Lyme disease (LD) occur more frequently when less stringent diagnostic criteria are used and that infections with *Borrelia burgdorferi* (Bb) may even cause or trigger primary dementia, such as Alzheimer’s disease (AD) [2–4]. The aim of this study was to characterize the clinical picture of dementia-like syndromes in definitive LNB, thus raising the awareness of this rare disorder. This should result in an early diagnosis and successful antibiotic treatment, as long as clinical symptoms are still completely reversible.

## Material and methods

Between 2004 and 2017, a total of 3 patients with a dementia-like syndrome and definite LNB were diagnosed at the neurological and at the psychiatric department of SMZ-Ost-Donauspital, a tertiary health facility. All patients fulfilled the EFNS guidelines for definitive LNB. Before LNB was diagnosed the clinical picture was consistent with the Diagnostic and Statistical Manual of Mental Disorders (DMS) IV criteria for dementia [5] and with the DMS-5 criteria for severe cognitive impairment, which have replaced the term dementia [6]. This article reports data from one of the three patients in detail. Data from the other two patients have been previously reported [7, 8]. For these two cases follow-up data will be reported.

To evaluate the frequency of this rare manifestation of LNB, patient files from 1 January 2004 to 31 December 2017 were searched at the neurological department of SMZ-Ost-Donauspital for all cases with LNB. In addition, a PubMed search was performed using the following keywords: “Neuroborreliosis” AND “Dementia”, “Neuroborreliosis” AND “Cognitive impairment”, “Neuoborreliosis” AND “Alzheimer’s disease”, “Neuroborreliosis” AND “Alzheimer”, “Neuroborreliosis” AND “Normal pressure hydrocephalus”. “Lyme disease”, and “Lyme Borreliosis” were also used as keywords instead of “Neuroborreliosis”. A total of 119 hits were retrieved. From these and other publications those 10 patients with detailed clinical information were selected. Of these 10 patients 2 with additional HIV positivity [9] and 1 patient with progressive supranuclear paralysis were excluded [10]. Cases which did not fulfil EFNS guidelines for definitive LNB [2, 11, 12], cases with definitive LNB and dementia, that did not present enough data for the individual patient [13, 59] and cases with cognitive impairment of less than 2 months duration [14, 15] were not included. Using diagnostic guidelines valid for European LNB our review was restricted to European cases with LNB.

## Case reports

**Fig. 1:**
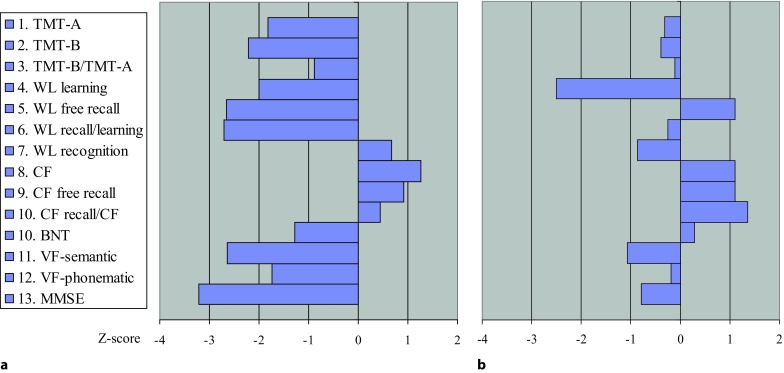
Patient 10, CERAD-Plus test profile **a** 4 months prior to antibiotic treatment and **b** 18 months after antibiotic treatment. *BNT* Boston naming test, *CF* copy figures, *MMSE* Mini Mental State Examination, *TMT* trail making test, *VF* verbal fluency, *WL* word list, z‑score (corrected for age, gender and educational level): negative values = worse

**Fig. 2:**
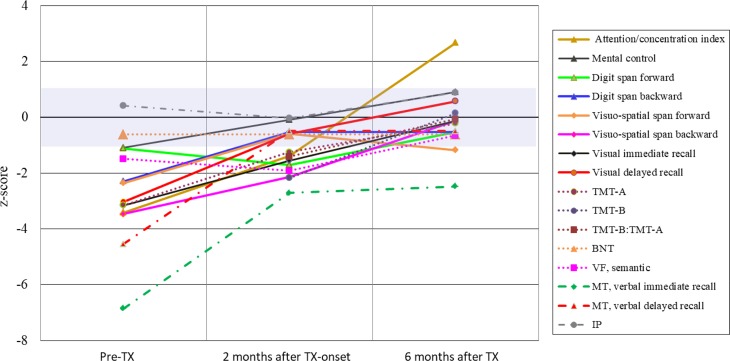
Patient 10, neurocognitive assessment. *Solid lines*: WMS-R subtests; *round dots*: CERAD-Plus subtests: *BNT* Boston naming test, *TMT* Trail making test, *VF* verbal fluency; dash-dot lines: *IP* ideomotor praxia, *MT* Memo test TX: antibiotic treatment; violet field: normal range (z-score −1 to +1); TMT-B z‑score for Pre-TX not shown, calculation due to delayed performance (598 s) not meaningful

**Fig. 3:**
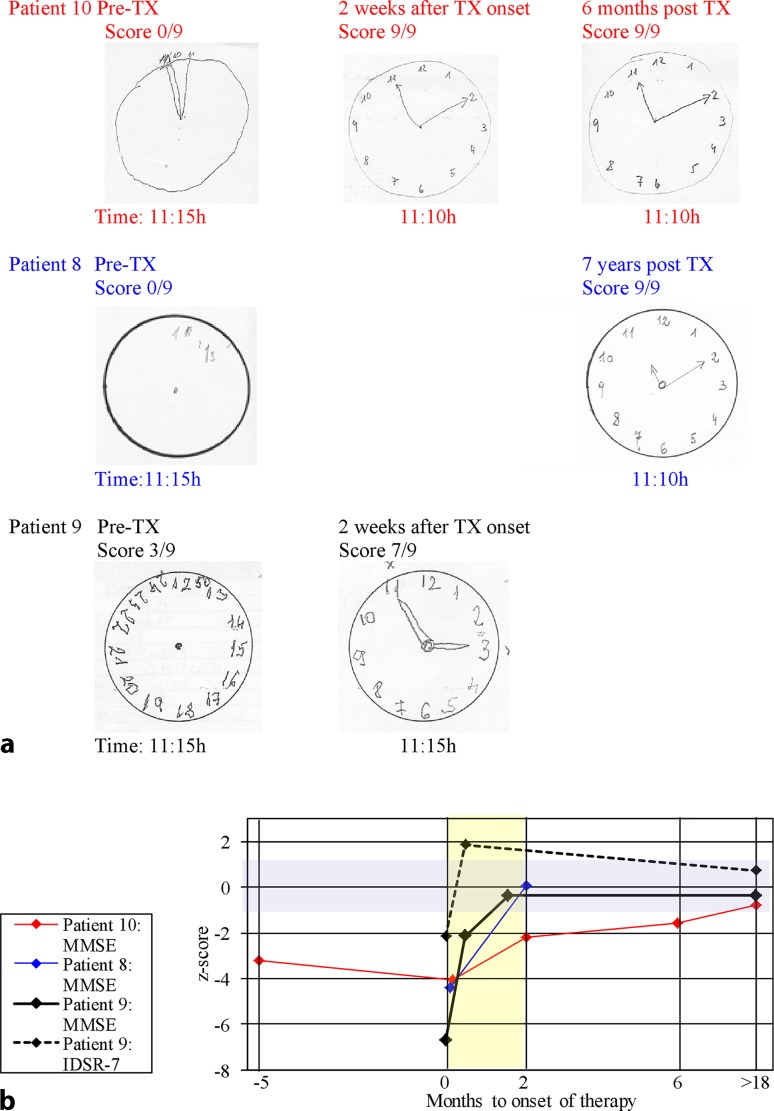
Clock-drawing test (**a**), Mini Mental State Examination test and Intercategorical Delayed Selective Reminding test (**b**) before and after antibiotic therapy. *IDSR* Intercategorical Delayed Selective Reminding test, *MMSE* Mini Mental State Examination test, *TX* antibiotic treatment, *violet field*: normal range (z-score: −1 to +1), *yellow field*: first 2 months after antibiotic therapy

**Fig. 4:**
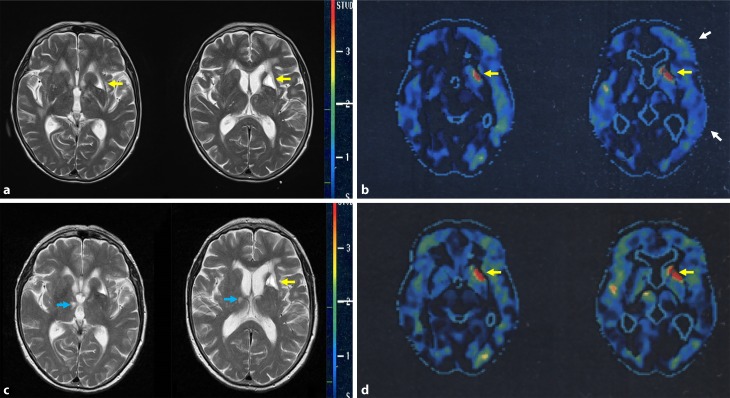
Patient 10, neuroimaging. MRI (T2-weighted images) 10 days before (**a**) and 9.5 months after antibiotic therapy (**c**). FDG-PET 2 months before (**b**) and 9.5 months after onset of antibiotic therapy (**d**). Metabolism evaluated by FDG-PET is presented as comparison with age matched healthy controls. Standard deviation is displayed with a red to dark blue scale. *White arrows* show hypometabolism in the left frontotemporal region before therapy (**b**) which is absent after therapy (**d**). *Yellow arrows* show left striatal lacunar lesion (**a** and **b**), not reversible after therapy (**c** and **d**). *Blue arrow* show small right thalamic vascular lesion (**c**), not seen in (**a**)

Patient 10 (Tables 1 and 2), a 76-year-old female, was referred to the department of neurology in July 2012 because of progressing cognitive decline over the last 12 months, loss of weight, nausea, gait disturbance and tremor. She was seen on May 2011 for the first time by a neurologist with a 3-month history of dull holocephalic headache who ordered a cranial magnetic resonance imaging (MRI) and diagnosed a tension-type headache and a depressive disorder. Treatment with an antidepressant (duloxetine) was started. The patient experienced no improvement and a second examination by another neurologist was undertaken 2 months later. Again no focal neurological signs could be detected. Due to the weight loss, an occult neoplasm was suspected but not detected during an extensive inpatient investigation at a medical department during February 2012; however, the MRI showed bilateral white matter lesions (WML) and an old lacunar lesion located at the left striatum, the latter was not seen in the previous MRI from May 2011. Since the patient also suffered from mild hypertension, vascular encephalopathy was thought to be the cause of the progressive cognitive decline. Extensive neurocognitive testing was carried out in a rehabilitation centre in May 2012 and disclosed a severe decline of attention, memory and executive functions corresponding to subcortical dementia (Fig. 1). When the patient was seen for a further diagnostic work-up at the SMZ-Ost-Donauspital in July 2012, the weight was 47 kg and a weight loss of 20 kg was reported over the past year. The gait was insecure with postural instability and with a tendency to fall when turning around. Frontal signs were positive, the voice was quiet, the tonus was mildly elevated and showed a slight hesitancy (“Gegenhalten”), tendon reflexes were brisk, paresis and pyramidal signs missing. There were no signs of ataxia, but a mild bradykinesia. Action tremor was more distinct than a mild resting tremor. Again, neurocognitive testing and gait disturbances were consistent with subcortical dementia (Figs. 2 and 3). Regarding the mild signs of parkinsonism, dementia with Lewy bodies (DLB) was also suspected but excluded by a dopamine transporter (DAT) scan. Fluorodeoxyglucose positron emission tomography (FDG-PET) demonstrated hypometabolism in the left striatum and in the left frontotemporal cortex (Fig. 4). Cerebrospinal fluid (CSF) showed signs of a chronic lymphocytic inflammation. The CSF markers for dementia, total tau protein and phosphor-tau were within the normal range, while beta-amyloid 1-42 and the Innotest-amyloid-tau index (IATI) were found to be below the reference values (beta-amyloid 1-42: 290 pg/ml, reference value > 500 pg/ml; IATI 0.6, reference values > 1). Finally, LNB was diagnosed when further CSF examinations disclosed a highly elevated Bb-specific-AI indicating local intrathecal Bb-specific antibody synthesis (Table 2). The patient was treated with 2 g ceftriaxone daily for 3 weeks.

**Table 1:** Clinical- and treatment-data of 10 patients with dementia like syndromes due to Lyme neuroborreliosis. Literature search and own cases

(Patient number), age at diagnosis (years), sex, (reference)	History of tick bite, EM or BS	Duration of symptoms prior to TX	Weight-loss	Nausea, malaise, vomiting	Head-ache	Voiding dysfunction	Tremor	Falls, gait disturbance	Other focal neurological signs	Neuroimaging	STT response
(1) 60, male [53]	EM? 12 m^a^ BS? 11 m^a^	6 m	Nm	Nm	Nm	+	Nm	+	No	cCT: normal	Nm
(2) 74, female [54]	No	8 m	Nm	Nm	Nm	+	Nm	+	No	MRI: ventricular dilatation, patch-like subependymal signal abnormalities, compatible with NPH	Yes/no
(3) 33, male [55]	No	8 m	Nm	Nm	Nm	Nm	Nm	+	Pyramidal signs	MRI: small hyperintense lesions close to cornu anterior and capsula externa	Nm
(4) 76, male [56]	Nm	6 m	+	Nm	Nm	+	Nm	+	No	MRI: ventricular dilatation suggesting NPH	Nm
(5) 83, female [57]	Repeated tick bites	6 m	5–7 kg	Nm	Nm	+	+	+	Diplopia	MRI: leukoaraiosis, ischemic lesion near left N lentiformis, enlarged ventricles suspicious for NPH	Yes/yes
(6) 69, female [39]	Remote tick bites	12 m	Nm	Nm	Nm	+	Nm	+	Babinski, leg weakness	MRI: symmetrical WML, meningeal GAD enhancement	Nm
(7) 75, female [58]	No	10 m	Nm	+	+	+	Nm	+	Rigor, brady-kinesia	MRI: mild periventricular white matter changes, widening of the lateral ventricles, Evans index 0.34	CSF < 10 ml
(8) 80, female [7]	No	6 m	Nm	Nm	Nm	+	+	(+)	No	MRI: enlarged ventricles, periventricular lesions	Yes/yes
(9) 71, female [8]	Tick bite EM ? 4 m^a^	3 m	15 kg	+	+	+	+	+	No	MRI: ventricles enlarged, bilateral mesiotemporal atrophy, widened insular cistern, cella media index 3.4. FDG-PET: normal	No
(10) 77, female	No	12 m	20 kg	+	+	Nm	+	+	No	MRI: bilateral WML, striatal lacunar lesion. FDG-PET: frontotemporal hypo-metabolism	No

*BS* Bannwarth’s syndrome, *cCT* cranial computed tomography, *EM* erythema migrans, *FDG-PET* fluorodeoxyglucose positron emission tomography, *GAD* gadolinium, *MRI* magnetic resonance imaging, *N* nucleus, *Nm* not mentioned, *NPH* normal pressure hydrocephalus, *STT* spinal tap-test

^a^Prior to antibiotic treatment

**Table 2:** Clinical- and treatment-data of 10 patients with dementia like syndromes due to Lyme neuroborreliosis. Literature search and own cases

(Patient number), reference TX, follow-up	Pre-/post-TX: cognitive impairment, MRI	MMSE	Pre-/post-TX: other neurocognitive tests	Pre-/post-TX: CSF
(1), [53]	Loss of memory and orientation in time, unable to cope with daily activities	Nm	Nm	Cc: 285/µl; tp: 3600 mg/l
2 w benzylpenicillin IV	Mental condition improved, memory poor, needs daily help (1 year after TX)	Nm	Nm	Cc: 88/µl; tp: 700 mg/l AI: 20
(2), [54]	Reduced attention and memory, confused, completely dependent	20/30	Digit-symbol (WAIS): 3; CAT: phasic and tonic alertness at least 1 sd below controls	Cc: 89/µl; tp: 1910 mg/l; OCB+; AI: 12.6
18 m after 2 w c	Memory normal, independent; MRI: idem	29/30	Digit symbol: 11; CAT: >1 sd above controls	Cc: 2/µl; tp: 290 mg/l; AI: >12.5
(3), [55]	Progressing impairment of memory and concentration	Nm	Nm	Cc: 51/µl; tp: 260 mg/l; OCB+; AI+
9 m after start of 2 w c	Major regression of cognitive impairment	Nm	Nm	Cc: 6/µl; AI+
(4), [56]	Amnesia for recent events, disorientation	15/30	Mattis Scale 98/144	Cc: 250/µl; tp: 3000 mg/dl (sic); AI: 19.7
4 w after start of 4? or 12? w c	No cognitive impairment after reassessment; MRI: unchanged after 1 m	“No impairment in neurocognitive tests”		“CSF normal” (12 w after start of TX)
(5), [57]	Impairment of memory and “wordfinding”	18/30	CERAD: impairment of vf and recall of world list	Cc: 69/µl; tp: 3542 mg/l; lactate: 4.8 mmol/l; AI: 31.1
7 m after 2 w c followed by 3 m a	No memory problems, no problems with daily activities	27/30	CERAD: vf, recall of world list improved	Nd
(6), [39]	Rapidly progressing dementia, short-term memory severely impaired, disoriented	Nm	Nm	Cc: 44/µl; alb: 3570 mg/l; lactate: 6.1 mmol/l; OCB-; AI: 10.5
5 m after 3 w c	No signs of cognitive impairment; MRI: improvement after 5 y	Nm	Nm	Alb: 244 mg/l; AI: 581
(7), [58]	Not fully oriented, attention, concentration and short-term memory reduced	20/30	Nm	Cc: 30/µl; tp: 1540 mg/l; lactate: 2.9 mmol/l; OCB+; AI: 18.5
a) 3 w, b) 4 m and c) 15 m after 3 w c	a), b) and c): complete remission; MRI: unchanged after 15 m	a) 28/30 b) 30/30 c) 30/30	Nm	Cc: a) 19 b) 3/µl; tp: a) 540 b) 390 mg/l lactate: a) 1.9 b) 1.6 mmol/l OCB (a and b)+; AI: a) 21.1: b) 49.9
(8), [7]	Reduced attention and memory, amnesia for recent events, spatiotemporal disorientation	21/30	Nm	Cc: 45/µl; tp: 523 mg/l; OCB+; AI: 13.6
2 m after start of 4 w c	Complete recovery; MRI: unchanged	29/30	Nm	Cc: 7/µl; tp: 370 mg/l; OCB+; AI: 10.9
(9), [8]	Spatiotemporal disoriented, reduced attention and memory, optic hallucinations	17/30	IDSR-5: −3.51, IDSR-7: −2.149 (z-score); CDT: 3/9	Cc: 321/µl; tp: 2351 mg/l; OCB+; AI: 7.0
(a) 11 d, (b) 12 m after start of 2 w c	a) Improvement in all neuropsychological parameters; b) stable	a) 27/30 b) 29/30	a) IDSR 5: +0.733, IDSR 7: −0.280 (z-score); a) CDT; 7/9	Nd
(10) case report	Attention-, memory-and executive deficits	22/30	CERAD, WMS-R, MT, CDT	Cc: 61/µl; tp: 3690 mg/l; OCB+; AI: 7.4
6 w (CSF), 6 m after 3 w c	Major improvement; MRI not improved	28/30	Improvement (see Figs. 1, 2 and 3)	Cc: 17/µl; tp: 1792 mg/l; OCB+; AI: 14.1

*a* amoxicillin 3 × 500mg/die orally, *AI* *Borrelia burgdorferi*-specific antibody index, *Alb* albumin, *c* ceftriaxone 2 g/die intravenously, *CAT* computerized alertness test, *Cc* cell count, *CDT* Clock-drawing test, *CERAD* Consortium to Establish a Registry for Alzheimer’s Disease test, *CSF* cerebrospinal fluid, *d* days, *IDSR* Intercategorical Delayed Selective Reminding test, *IV* intravenously, *m* months, *MMSE* Mini Mental State Examination test, *MRI* magnetic resonance imaging, *MT* Memo test, *Nd* not done, *Nm* not mentioned, *OCB* oligoclonal banding, *sd* standard deviation, *tp* total protein, *TX* antibiotic treatment, *vf* verbal fluency, *w* weeks, *WAIS* Wechsler Adult Intelligence Scale, *WMS-R* Wechsler Memory Scale-Revised, *y* year

Neurological symptoms and impaired cognitive functions, although persistent for a year, recovered rapidly within a few weeks (Figs. 1, 2 and 3) and so did the pathological CSF findings (Table 2). A follow-up FDG-PET examination showed the left frontotemporal hypometabolism in remission, while this was not the case for the cystic lacunar lesion in the left striatum. A new and clinically silent small right thalamic lesion was detected that was not present in the pretreatment MRI (Fig. 4). The Consortium to Establish a Registry for Alzheimer’s Disease (CERAD) test battery at the last follow-up in April 2014 scored within the age-dependent normal range with the exception of verbal learning and semantic verbal fluency (Fig. 1). In a telephone call in February 2018 at the age of 82 years, the patient reported no gait problems or cognitive impairment and had just returned from a trip to Cuba.

Patient 8 (Table 1 and 2), an 80-year-old female, was admitted to hospital in Mai 2006 because of gait disturbances, cognitive decline and frequent falls. The cranial computed tomography (cCT) showed enlarged ventricles and NPH was initially suspected. During a spinal tap test (STT) for predicting response to shunting, the CSF unexpectedly showed signs of an aseptic meningitis and LNB was revealed. The symptoms resolved completely after antibiotic treatment with ceftriaxone 2 g daily for 4 weeks (Table 2; Fig. 3b; for more details see reference [7]).

The patient had a follow-up visit in 2013 when living independently in a retirement home, was fully ambulatory, oriented and showed no signs of cognitive impairment in the Clock-drawing test (CDT) (Fig. 3a).

Patient 9 (Tables 1 and 2), a 71-year-old female, was admitted to the psychiatric department of our hospital in November 2010 with the initial diagnosis of rapidly progressing dementia or delirium. A history of mild forgetfulness which was noticed half a year prior to the beginning of rapid deterioration and a slight mesiotemporal atrophy in the MRI together with a pathological score in the Mini Mental State Examination (MMSE) test and in the Intercategorical Delayed Selective Reminding Test (IDSR) supported the initial diagnosis of primary dementia (Table 2; Fig. 3). Short periods of altered consciousness on admission were compatible with a delirious state. Later, the patient’s daughter reported a tick bite followed by a widespread rash. Thus, LNB was suspected and confirmed by CSF investigations (Table 2). The patient’s cognitive impairment remitted within the 2 weeks of antibiotic treatment with 2 g ceftriaxone/day (Fig. 3). When discharged from the hospital the patient was still on galantamine and mirtazapine (for more details see reference [8]).

At the first follow-up investigation after 1 month the patient scored 29/30 (z −0.375) in the MMSE and treatment with galantamine was stopped. At the second follow-up 1 year after treatment of LNB cognition was normal with MMSE 29/30, and mirtazapine could be stopped. At another follow-up 5 years and 5 months after treatment of LNB, cognition was stable, and testing of episodic memory by the IDSR 7 now showed a z-score of +0.734, i.e. above the mean for females of the same age, which strongly argued against any dementing process (Fig. 3b).

## Reports from the literature

The literature search by using stringent diagnostic criteria disclosed only seven additional patients with dementia as a leading symptom of definitive LNB. These cases were reported in detail, so they can serve for the description of characteristic features. The data of all 10 patients are summarized in Table 1 and 2.

## Frequency of dementia-like syndromes among patients with Lyme neuroborreliosis

In a search for adult inpatients with the diagnosis LNB at our neurological department over a period of 14 years, 48 other patients with definitive LNB were identified. The vast majority (*n* = 45) were diagnosed with Bannwarth’s syndrome (BS), a well characterized painful radiculoneuritis. Subacute meningoencephalitis (*n* = 1), facial palsy without other symptoms of BS (*n* = 1) and acrodermatitis chronica atrophicans-associated neuropathy (*n* = 1) were other rare diagnoses. Patient records prior to 2004 were not available for the search, but the authors do not remember having seen patients with dementia-like syndromes and chronic LNB during the 20 years previous to 2004, a period when serodiagnostic tests were already available. Thus, the frequency of patients with dementia-like syndromes amongst adult patients with LNB in the setting of a tertiary health facility was well below 6%.

## Discussion

The cases with secondary dementia reported here are manifestations of chronic progressing meningoencephalomyelitis in LNB, originally reported by Ackermann in 1985 [16]. This rare disorder is caused by an active and ongoing CNS inflammatory process and occurs in about 2–6% of all cases with LNB [17–19]. It must not be mistaken for Lyme encephalopathy [20], “pure Lyme dementia” [2] or for neuropsychological complaints of the “post Lyme disease syndrome”, conditions without proof of an ongoing CNS infection [21]. The largest case series of this rare disorder includes 44 patients and shows a broad clinical spectrum. Spastic paraparesis or tetraparesis, ataxia, incontinence, dysarthria and hearing impairment were the most frequent symptoms. While mild impairment of memory and concentration was seen in 12 of the 44 patients, 2 patients “disclosed severe mental disorders with dementia-like deficiencies, loss of orientation and even altered consciousness” [59]. In a few cases of CNS-LNB, cognitive impairment was so dominant that they were initially misdiagnosed as rapidly progressing primary dementia. A joint presentation of these cases may be helpful to avoid diagnostic pitfalls. In the following, clinical features which raise suspicion of LNB in dementia-like syndromes are outlined.

### History

Previous tick bites, EM or symptoms compatible with early LNB like BS were remembered only by a small minority of patients with chronic LNB or by their relatives. When patients did remember, chronic progressive encephalomyelitis followed 2 months to 2 years later [22, 23]. Thus, EM or other characteristic symptoms of early LB 1–2 years before the onset of dementia may if untreated serve as an indicator for chronic LNB.

### Disease course

Dementia in LNB showed a rapid course reaching a moderate stage of dementia within 6–12 months from onset of symptoms. Aside from dementia in Creutzfeldt-Jakob disease which occurs rapidly, most neurodegenerative dementias develop slowly with the possible exception of DLB and corticobasal degeneration. Disorders that commonly lead to a slowly progressive dementia such as AD and frontotemporal dementia rarely present with a rapid course [24]. Cognitive impairment with sudden onset or with stepwise deterioration in combination with hemiparesis, hemianopsia or diplopia indicating vascular dementia [25] have also been reported in rare cases of vasculitis due to LNB and might be the cause of irreversible dementia in LNB [26–28].

### Somatic symptoms

Weight loss is another symptom observed in LB. It is also compatible with the diagnosis of AD [29] but when it occurs in chronic LNB, it can be more pronounced, reaching up to 20 kg/year [12, 22, 30–32], while weight loss, which is seen in almost all AD patients is less prominent and is on average 2 lb (0,9kg)/year [33].

Headache, nausea, malaise and vomiting, probably signs of chronic meningitis, are not symptoms of degenerative dementias but might be associated with secondary dementia and thus also with chronic LNB as was the case in 2 of our 3 patients. Sensorineural hypacusis, another symptom associated with chronic meningitis and not recorded in our patients, was a characteristic symptom in other cases of chronic CNS-LNB [17]. Tremor was reported in all three of our patients, in two of them as action tremor. In the third patient, who showed resting and position tremor, the latter dominated.

Gait disturbances at the onset or early in the disease which was observed in all cases of this study, makes the diagnosis of a probable AD uncertain or unlikely [29]. It presents as postural instability, ataxia or as broad-based, short-stepped gait. A walker had to be used by two out of three and falls were reported by all three of our patients. Progressive gait disturbance in combination with voiding dysfunction and dementia forms the clinical triad of NPH symptoms [34] and in 5 of the 10 patients probable NPH was the initial diagnosis (patients 2, 4, 5, 7 and 8 in Table 1). When they had a STT, performed for predicting response to shunting, CSF pleocytosis was unexpectedly detected and further CSF analyses disclosed the correct diagnosis of secondary NPH due to LNB. Secondary NPH was also seen in several other cases of LNB not included in this study [13, 17, 30, 35, 36]. Results of STT were reported in four patients (Table 2 and reference [30]) and two of them showed improvement of symptoms. Details on the improvement evaluation were missing. Of the 68 patients with LNB reported from a retrospective data analysis 5 had chronic LNB. All 5 had symptoms indicative of NPH, but lumbar puncture with drainage of CSF did not change the symptoms [13]. Furthermore, the clinical symptoms between patients with and without MRI signs of NPH did not significantly differ in our patients. Whether CSF absorption difficulties, CNS inflammation or the combination of both, were the cause of the cognitive decline in these cases remains a matter of discussion [7].

### Neuropsychological assessment

Results of the MMSE reported in 7 of the 10 patients were in accordance with mild (*n* = 3) to moderate (*n* = 4) dementia. All three of our patients had a low score in the categories recall, orientation and complex commands, and two of the three also in the category attention. Scores in the CDT, another global screening test for dementia, also showed signs of marked cognitive impairment in all three patients (Fig. 3). Patients 10 and 9 had additional tests. In patient 9, episodic memory investigated with the IDSR was significantly reduced, but 11 days after onset of antibiotic therapy, MMSE, CDT and IDSR scored already in a range no longer consistent with dementia. Patient 10 had extensive neuropsychological testing including subtests from CERAD and Wechsler Memory Scale-Revised (WMS-R) test battery. The results showed deficits involving memory, attention, psychomotor speed, orientation and executive functioning, altogether consistent with subcortical dementia. Deficits of verbal memory retrieval by spared verbal recognition found in this patient, speak against primary dementia and AD but for secondary memory impairment and are a strong indicator for subcortical dementia [37]. Similar results have been seen in the only other patient also tested with a CERAD test battery (patient 5, Table 2).

### Neuroimaging

Mild ventricular enlargement was seen in 6 of the 10 patients. Different WML, patch-like, symmetrical confluent, periventricular subependymal or lacunar, were reported. Lacunar lesions in chronic LNB are most likely due to small and medium-sized meningeal vessel vasculitis, as disclosed by autopsy [38]. Of the 9 patients with MRI examinations one had no WML at all. Patient 6 with initially large symmetrical WML that were similar to those seen in patient 10, also showed meningeal gadolinium enhancement. In a MRI follow-up 5 years later, surprisingly, most of the original lesions had resolved [39]. The MRI results remained unchanged in the other case reports with follow-up investigations reported. This may be due to the shorter follow-up intervals of 18 months or less. Of the patients two had FDG-PET examinations: one patient with suspected AD and no MRI-WML had a normal glucose metabolism (patient 9 Table 1), the other, who had bilateral large WML and a lacunar lesion in the left striatum in the MRI examination, showed pathological FDG-PET results. In addition to the expected hypometabolism corresponding with the lacunar lesion, hypometabolism in a left cortical frontotemporal region was also detected. The latter was reversible in a posttreatment follow-up investigation and might have been due to hypoperfusion. The small additional thalamic lesion which was detected in an MRI follow-up (Fig. 4) did not correspond to the continuing clinical improvement. Whether it developed shortly before or during the treatment period or later remains unclear. Altogether, results of MRI and FDG-PET examinations did not show a uniform pattern.

### Treatment

Results of antibiotic treatment in 9 of 10 patients were excellent, even in those 3 patients with only a 2-week course of ceftriaxone. One patient treated with intravenous benzylpenicillin for 2 weeks continued to need supervision and help. In most patients improvement of symptoms was reported already within few days of antibiotic treatment. This was also the case in the patient with prolonged antibiotic treatment who received 2 weeks ceftriaxone followed by oral amoxicillin for 3 months (patient 5, Table 2). The CSF pleocytosis, elevated total protein and elevated lactate improved parallel to the clinical remission, while OCB and AI persisted. Persisting AI and OCB in otherwise normal CSF are frequently seen in adequately treated LNB and should not be interpreted as an ongoing active infection [40]. Relapses or ongoing worsening were not observed. Altogether, there was no indications for a prolonged antibiotic therapy in our patients.

### Pathogenesis

The pathogenesis of chronic encephalomyelitis in LNB is not yet fully understood. Focal inflammation, demyelination, vasculitis with subsequent ischemic lesions and autoimmune phenomena have been thought to play a role. Only very few pathological studies on CNS specimens exist and described leptomeningitis, vasculitis and focal inflammation in the CNS [38, 41]. Animal models of LNB have demonstrated that invasion of the CNS with *Borreliae* induces a strong, cytokine-driven inflammatory reaction. This causes pathological alterations similar to those seen in human LNB. Dexamathasone prevented these pathological alterations although *Borelliae* persisted [42]. This could explain the alternating disease course with a tendency to improvement in a case report on chronic CNS-LNB, which was treated with corticosteroids and azathioprine for 2.5 years. Finally, the correct diagnosis was established and the patient had a full remission after a 2 weeks course of 3 × 5 million units iv penicillin G/day [43]. Secondary NPH reported in half of the patients, may also play an additional role in the development of dementia-like syndromes.

## Conclusion

Dementia-like syndromes in LNB belong to the broad clinical spectrum of chronic CNS-LNB, an active and ongoing inflammatory disorder of brain, spinal cord, meninges and leptomeningeal vessels. The clinical manifestations of this rare disorder depend on the neurological structures which are primarily involved. The full clinical picture of progressive encephalomyelitis in LNB may mimic chronic progressing multiple sclerosis [16]. Cases with predominant meningeal involvement have been mistaken for tuberculous meningitis [44]. Other rare cases present as extrapyramidal [45, 46] or as psychiatric disorders, such as schizophrenia-like psychosis [47–50], catatonia [51] or mania [52]. In all cases with dementia-like syndromes reported in our review, LNB was originally not taken into consideration. This justifies the aim to point to characteristic signs and symptoms of this rare manifestation of chronic CNS-LNB. The detection should result in CSF examinations including determination of Bb-specific AI which discloses the correct diagnosis. Even in chronic LNB, early antibiotic treatment is essential and can prevent irreversible sequelae.

## Abbreviations

AD: Alzheimer’s disease
AI: *Borrelia burgdorferi* specific antibody index
Bb: *Borrelia burgdorferi*
BS: Banwarth’s syndrome
cCT: Cranial computed tomography
CERAD: Consortium to Establish a Registry for Alzheimer’s Disease
CDT: Clock-drawing test
CNS: Central nervous system
CSF: Cerebrospinal fluid
DAT: Dopamin transporter
DLB: Dementia with Lewy bodies
DMS: Diagnostic and Statistical Manual of Mental Disorders
EFNS: European Federation of Neurological Societies
EM: Erythema migrans
FDG-PET: Fluorodeoxyglucose positron emission tomography
IATI: Innotest-amyloid-tau index
IDSR: Intercategorical Delayed Selective Reminding test
LB: Lyme borreliosis
LD: Lyme disease
LNB: Lyme neuroborreliosis
MMSE: Mini Mental State Examination
MRI: Magnetic resonance imaging
NPH: Normal pressure hydrocephalus
STT: Spinal tap test
WML: White matter lesions
WMS-R: Wechsler Memory Scale-Revised
